# Analysis of early changes in DNA methylation in synovial fibroblasts of RA patients before diagnosis

**DOI:** 10.1038/s41598-018-24240-2

**Published:** 2018-05-09

**Authors:** Emmanuel Karouzakis, Karim Raza, Christoph Kolling, Christopher D. Buckley, Steffen Gay, Andrew Filer, Caroline Ospelt

**Affiliations:** 1 Center of Experimental Rheumatology, Department of Rheumatology, University of Zurich, Zurich, CH-8952 Switzerland; 2 Institute of Inflammation and Ageing, University of Birmingham, Edgbaston, B15 2TT UK; 3 Schulthess Clinic, Zurich, CH-8008 Switzerland; 4 Sandwell and West Birmingham Hospitals NHS Trust, West Bromwich, B71 4HJ UK; 5 University Hospitals Birmingham NHS Foundation Trust, Birmingham, B15 2 GW UK

## Abstract

DNA methylation is an important epigenetic modification that is known to be altered in rheumatoid arthritis synovial fibroblasts (RASF). Here, we compared the status of promoter DNA methylation of SF from patients with very early RA with SF from patients with resolving arthritis, fully established RA and from non-arthritic patients. DNA was hybridized to Infinium Human methylation 450k and 850k arrays and differential methylated genes and pathways were identified. We could identify a significant number of CpG sites that differed between the SF of different disease stages, showing that epigenetic changes in SF occur early in RA development. Principal component analysis confirmed that the different groups of SF were separated according to their DNA methylation state. Furthermore, pathway analysis showed that important functional pathways were altered in both very early and late RASF. By focusing our analysis on CpG sites in CpG islands within promoters, we identified genes that have significant hypermethylated promoters in very early RASF. Our data show that changes in DNA methylation differ in RASF compared to other forms of arthritis and occur at a very early, clinically yet unspecific stage of disease. The identified differential methylated genes might become valuable prognostic biomarkers for RA development.

## Introduction

Rheumatoid arthritis (RA) is a chronic, destructive autoimmune arthritis that affects 0.5–1% of the population worldwide. The development of RA begins long before patients present with the characteristic symptoms of RA. Many patients develop unspecific symptoms and signs of systemic autoimmunity i.e. auto-antibodies years before disease onset^1^. Even though these individuals have an increased risk of developing RA, not all of them do and it remains a challenge to discriminate patients at a very early stage of RA from patients with resolving arthritis.

Synovial fibroblasts are major contributors to joint inflammation and destruction in RA. Changes in DNA methylation have long been recognized in synovial fibroblasts of RA patients (RASF)^2–4^ and pathway analysis of differentially methylated sites (DMS) suggested that these changes affect genes previously implicated in the pathogenesis of RA^5^. Thus, it is assumed that changes in DNA methylation contribute to the activated and destructive phenotype of RASF.

DNA methylation involves a methyl group attached in the 5′ position of the cytosine base pair ring called 5-methylcytosine (5-mC). This chemical modification is catalysed by a family of enzymes called DNA methyltransferases.In humans, the enzymes DNMT1, DNMT3A and DNMT3B have been shown to methylate genomic DNA. Most methylation of DNA is found at CpG dinucleotides. Sequences that have high percentage of C and G nucletoides and have a length of 1000 bp constitute genomic regions called CpG islands. When these regions are methylated, they cause gene silencing. DNA methylation has been analysed in different disease such as fibrosis, cardiovascular and cancer^6^. A number of hypomethylated and hypermethylated regions have been found to be involved with disease pathogenesis. Recent research has shown that the 5-mC modification of DNA can be converted into 5- hydroxyl-methylcytosine (5-hmC), 5-formyl-cytosine (5-fC), 5-carboxyl-cytosine (5-caC), and reconverted into 5-mC through the activation of ten-eleven translocation family enzymes (TET1–3) and thymine DNA glycosylase (TDG)^7^. This mechanism of DNA demethylation and 5-hmC is associated frequently with active gene expression.

Two aspects in the analysis of DNA methylation in RASF, however, have not been addressed until now. Firstly, it is not known whether changes in DNA methylation are a cause or a consequence of the chronic inflammatory process in RA. Published studies so far have used SF from patients who have already fulfilled classification criteria for the diagnosis of RA; it is not known how early in the disease process changes of DNA methylation occur in SF. Secondly, current studies mostly analyzed differences in the DNA methylation profile of RASF compared to OASF. Synovial inflammation frequently occurs in OA and while not growing invasively like RASF, transcriptome studies comparing OASF with healthy SF and RASF show clear differences between healthy SF and OASF, indicating that OASF also display an altered phenotype^8,9^. Thus, it is difficult to estimate which changes in DNA methylation are RA specific and which are OA specific and it is unknown up to now how changes found in RASF compare to other inflammatory arthitides.

To address these questions we analyzed the DNA methylation profile of SF isolated from patients with total symptom duration of three months or less who fulfilled the 1987 ARA criteria by 18 months after the biopsy was taken. The DNA methylation profile of these very, early RASF (veRASF) was compared to SF isolated from healthy individuals experiencing transient joint pain without histological signs of arthritis (nSF) as well as to SF isolated from patients with transient, non-destructive arthritis of varying etiology, which resolved within 18 months (rSF) of biopsy taken. Furthermore, SF from patients with established, long-standing RA (estRA) were included. With this approach, very early changes of SF in the RA disease process compared to normal SF as well as to other types of arthritis could be identified.

## Results

**Figure 1:**
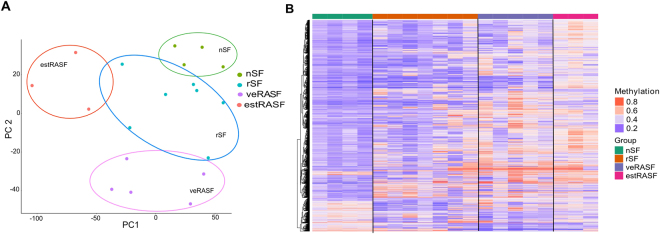
Differentially methylated CpGs at promoter sites (DMPs) differ between diseases and disease stage. DNA methylation at CpGs of promoter regions were compared between SF isolated from healthy individuals (nSF) and patients with resolving, acute arthritis (rSF), very early rheumatoid arthritis (veRA) and patients fulfilling the criteria for the diagnosis of RA (estRA), respectively. (**A)** Principal component analysis (PCA) showed a clear separation of SF based on DMPs between the different conditions. (**B)** Heatmap of beta values of DMPs across synovial fibroblasts of the different groups. Every row represents the beta value of a DMP CpG and every column is one patient. The colour refers to the level of DNA methylation as shown in the color key.

DNA methylation patterns of nSF were compared to DNA methylation of rSF, veRASF and estRASF. We focused our analysis on promoter regions, as promoter DNA methylation is most likely to have an impact on gene expression. Analysis of differentially methylated CpG sites in promoter regions (DMP) revealed 772 significantly differentially methylated CpGs between nSF and rSF, 1792 between nSF and veRASF and 2168 between nSF and estRASF (p < 0.05, delta-beta >0.1, Supplemental Table 2). PCA showed that DMPs separated SF according to both disease groups and disease stage (Figure 1A). During disease development from nRA to veRA and estRA, the total number of hypermethylated CpG sites in gene promoters increased from 9% of CpGs having beta values of >0.4 in nSF, to 84% in veRASF and 96% in estRASF. In contrast, in rSF only 22% of CpGs in promoter regions were hypermethylated (Figure 1B).

We narrowed down our analysis and specifically looked at DMP lying in genomic regions with a high content of CpGs (CpG islands) within promoter regions. These changes in DNA methylation are believed to directly affect gene transcription. For each disease stage, we found a specific set of CpG islands with differences in DNA methylation (Table 1). CpG islands with the promoters of peptidase M20 domain containing 1 (PM20D1), SHROOM1 and the homeobox protein engrailed-1 (EN1) were significantly hypermethylated in veRASF compared to nSF. In estRASF, another set of CpGs within promoters such as the microfibrillar associated protein 2 (MFAP2), the receptor kinase Epithelial discoidin domain-containing receptor 1 (DDR1) and the major histocompatibility complex HLA-C were hypermethylated. Interestingly, even though not reaching statistical significance in all conditions, a shift in the direction of DNA methylation from hypomethylation in normal towards hypermethylation in veRASF and estRASF was visible in PM20D1, MFAP2 and SHROOM1 (Figure– 2A,B Supplementary Figure 1A,B). We further confirmed that DNA methylation has a functional role in the gene expression of SHROOM1 by demethylating the estRASF with 5-azacytidine. Interestingly, we noticed a decreased in the methylation levels and upregulation of SHROOM1 expression in these cell cultures (Supplementary Figure 2A,B).

**Table 1:** Differentially methylated CpG islands within CpG promoters (DMP) with at least 3 differentially methylated CpG sites.

Gene	Island	Methylation	p.value
**nSF vs veRASF**			
PM20D1	chr1:205818898-205819191	hyper	0.019
EN1	chr2:119599458-119600966	hyper	0.025
SHROOM1	chr5:132158542-132159203	hyper	0.003
**nSF vs estRASF**			
MFAP2	chr1:17306551-17307363	hyper	0.0105
RIMBP2	chr12:130935712-130935963	hyper	0.0038
IRX6	chr16:55362737-55363287	hyper	0.0147
DDR1	chr6:30852102-30852676	hyper	0.0152
HLA-C	chr6:31238852-31240120	hyper	0.0192

**Figure 2:**
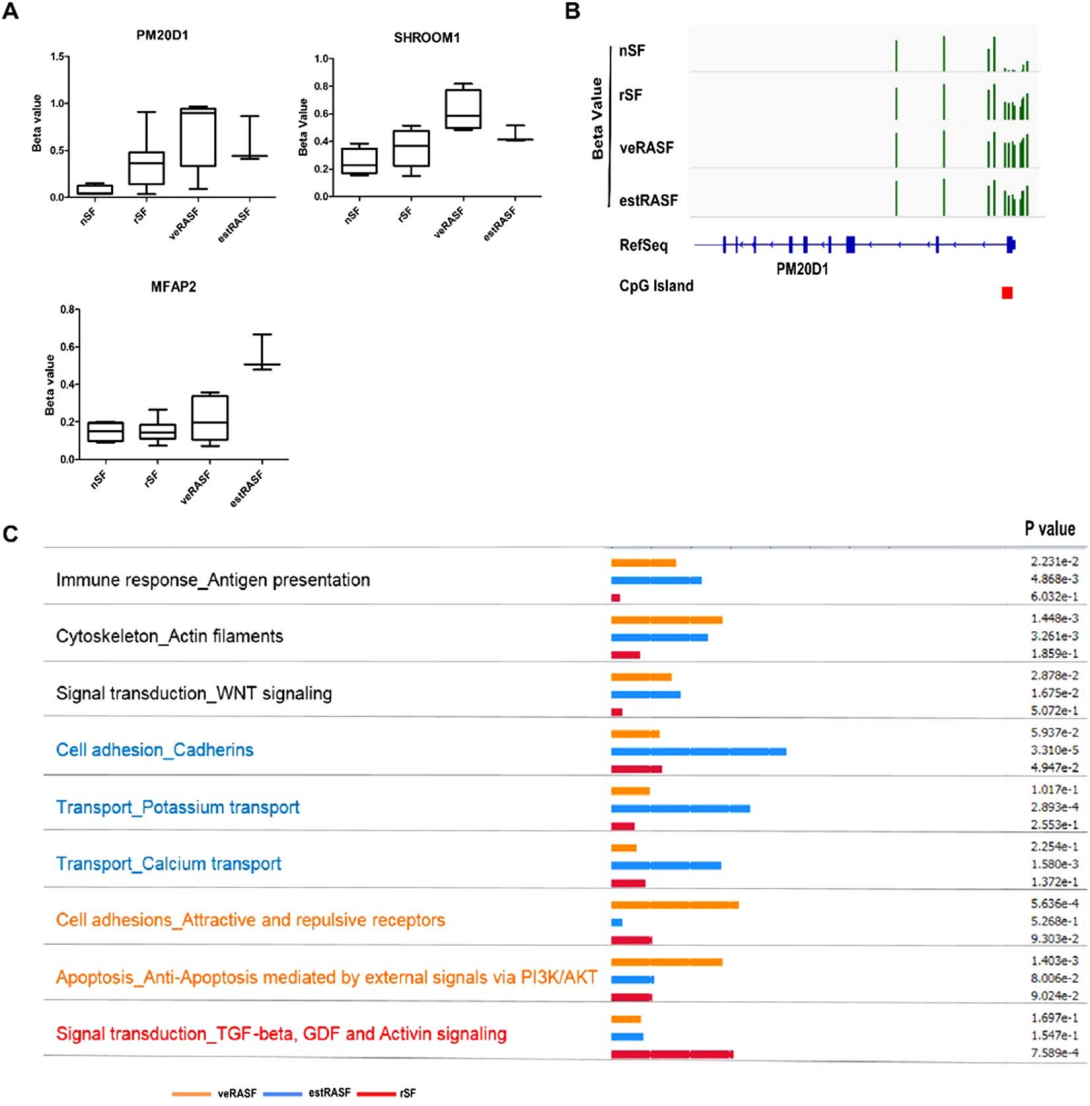
Hypermethylated genes and pathways between different disease stages. (**A**) An increase of the average beta values of PM20D1, MFAP2 and SHROOM1 occurred between nSF, rSF, veRASF and estRASF. The box plots show the media, minimum, maximum and quartiles of beta values. (**B)** Hypermethylation was observed in the CpG Island within the PM20D1 promoter of rSF, veRASF and estRASF. The graph represents DNA methylation beta values for each sample aligned to the genomic reference and CpG Island of hg19 UCSC genomic assembly by IGV viewer software. (**C**) The top enriched pathways in significantly differential methylated sites (DMS) in very early RASF (veRASF), established RASF (estRASF) and resolving arthritis (rSF) respectively are depicted.

Last, we used the individual DMS (Supplemental Table 2) and searched for biological pathways that potentially are altered during disease development caused by the identified changes in DNA methylation. Several pathways were commonly altered in veRASF and estRASF compared to nSF, but not significantly changed in rSF (Figure 2C). These pathways included cadherin, integrin and WNT cell adhesion signaling pathways, components of the actin cytoskeleton and antigen presentation.

Last, we measured the expression of TET1 and DNMT1 enzymes between the different disease groups in order to identify a potential mechanism for the hypermethylation of gene promoters in early disease. Interestingly, we found a significant increase in TET1 and DNMT1 in rSF in comparison to the normal group but not in the veRASF group (Supplementary Figure 3A,B). These results suggest an ongoing epigenetic reprogramming during resolution of the disease which is lacking in RA patients.

## Discussion

In the current study, we compared DNA methylation patterns between nSF and SF from different stages of RA and transient, resolving arthritis. In doing so, we obtained a detailed view of changes in DNA methylation that occur during the development of RA from the earliest clinically apparent stages to established RA with its typical signs and symptoms. Furthermore, we could identify changes prior to treatment with disease modifying agents that are specific for SF in a very early stage of RA and are not found in SF from patients with equivalent levels of inflammation but whose eventual outcome is the resolution of synovitis. These findings support the hypothesis that changes in DNA methylation in SF are not a mere consequence of a long-standing pro-inflammatory environment, but are initial drivers of the persistence of disease in RA.

Our data confirm findings of studies analyzing RA patients in this very early window of clinically apparent disease. In synovial fluids of RA patients of this stage, levels of several cytokines and growth factors were higher than in patients with other causes of arthritis and with established RA^10^. Similarly, increased levels of macrophage derived CXCL4 and CXCL7 were found to be elevated in synovial tissues of RA patients at this very early phase of disease^11^. In accordance with the current data, where changes in the Wnt signaling pathway were found, veRASF were shown to produce higher amounts of Dickkopf-related protein 1 (DKK-1), a member of the Wnt signaling pathway compared to patients with resolving arthritis^12^. Also in studies analyzing changes in DNA methylation of SF isolated from TNF transgenic mice at different time points and of patients with treated established early and late RA, the Wnt signaling pathway showed stage specific changes^13,14^. Most interestingly, DNA methylation in antigen presentation network processes was specifically altered in veRASF and estRASF, but not in rSF. SF are recognized as antigen presenting cells that can induce adaptive immune functions^15,16^. Our data strongly suggest that antigen presentation by SF is activated early in RA and thus might be a crucial contributor to the propagation and chronification of the autoimmune response in RA. Together, these data consistently demonstrate that despite presentation in very early or undifferentiated forms, distinct pathways already operate in the synovium of RA patients at very early stages.

Our analysis showed a shift towards hypermethylation of gene promoters in RASF compared to nSF. Previously, we observed global hypomethylation in SF from RA patients in late disease stages^4,17^ and we also detected global hypomethylation in veRASF (data not shown). It is important to stress that the experimental and bioinformatics tools, applied by us in the current study and by others in previous studies, are not suitable for measurements of global methylation. Global DNA hypomethylation as found in RASF mainly originates from a loss of DNA methylation in non-coding, repetitive and LINE retrotransposable sequences, which is not captured with methylation arrays^18^. Thus, similar to findings in cancer cells, RASF present with global DNA hypomethylation, but promoter-specific hyper and hypomethylation.

Our study identified several CpG islands that were specifically hypermethylated in SF from very, early and/or established RA, but not in resolving arthritis. MFAP2 (MAGP1) binds TGFβ and members of the bone morphogenic protein (BMP) family^19^, suggesting a role of MFAP2 in regulating the release and activity of these factors that were previously implicated in arthritis development. The enzyme PM20D1 recently has been shown to play a role in glucose homeostasis and energy expenditure by controlling the biosynthesis of lipid metabolites in the body^20^. SHROOM1 has been found differential methylated in knee chondrocytes of OA patients^21^ and was connected to microtubule rearrangements during cell elongation^22^. DDR1 tyrosine kinase was found to bind collagens^23^ and can regulate various cellular processes such as migration, invasion and cell proliferation. Overall, our data provide promising targets for further functional experiments that could elucidate the phenotypic changes of RASF and their invasive behavior.

More importantly, we identified a set of genes with substantial differences in DNA methylation between very early RA and resolving arthritis, in particular SHROOM1. These differences could serve as valuable biomarkers for early diagnosis of RA. Methylated DNA is very stable. It can been detected in archived clinical samples, such as paraffin embedded tissue samples and is already used as biomarker in clinical practice, for instance in glioblastoma patients^24^. Currently, studies are initiated to test the suitability of these sites to predict RA development in synovial biopsies of patients with undifferentiated arthritis.

In summary, this is the first study providing evidence that DNA methylation in RASF is changed in the very earliest stages of untreated RA. Furthermore, our data confirm several previous studies suggesting that cell adhesion, cytoskeletal rearrangement and alterations in the Wnt signaling pathway in SF play a role in RA disease development. Future studies will explore how such changes in DNA methylation are induced, how the identified genes and pathways contribute to the pathogenesis of RA and whether the changes in DNA methylation can be used as prognostic biomarkers for the development of RA.

## Methods

### Synovial Fibroblasts

Ultrasound guided biopsies were taken from patients recruited to the UK Birmingham early arthritis (BEACON) cohort^11^ after written informed consent and permission from the West Midlands Black Country Research Ethics Committee. In addition, we confirm that all the experiments were performed in accordance with the relevant guidelines and regulations. SF were isolated and cultured as previously described^25^. Normal SF were obtained from individuals experiencing transient joint pain without histological signs of arthritis. VeRASF and rSF were isolated from treatment naïve patients presenting with inflamed joints with a symptom duration of 3 months or less. Patients were retrospectively classified as having had veRA if they fulfilled the American Rheumatism Association (ARA) 1987 revised criteria for the classification of RA within 18 months of the biopsy taken^26^. EstRASF were isolated from patients fulfilling the 1987 ARA criteria at presentation. A detailed description of the patients is given in Supplemental Table 1.

### Illumina methylation arrays

DNA was isolated from cultured fibroblasts with the DNA blood kit (Qiagen). DNA was converted by sodium bisulfite modification and hybridized to Infinium Human methylation 450k and 850k arrays. The raw intensity data (IDAT) were imported into R version 3.4.0 and processed using the minfi (1.22.1) Bioconductor package. Since the methylation data sets were run on different Illumina array types, we combined the arrays at a probe level into a single 450k array with the minfi function ‘combine Arrays’ as previously published. All the samples passed the mean detection p-value of the minfi sample quality standard^27^. Preprocess normalization was performed using the ssNoob method. Probes from the X and Y chromosome were removed to exclude sex variability and probe type bias was corrected using the rcp method as described in the Enmix bioconductor package (1.12.0). Individual beta values for each sample were calculated for further statistical analysis. Beta values are defined as the ratio of methylated probe intensity and overall probe intensity (sum of both unmethylated and methylated probe intensity).

### Differential methylation analysis

The COHCAP algorithm was applied for the statistical analysis of differentially methylated CpG sites and islands^28^. A CpG site was counted as methylated if the beta value was greater than 0.4 and unmethylated if it was below 0.3. Differential methylated CpG sites (DMS) in promoter regions (DMP) and/or CpG islands that showed mean beta value differences of 0.1 and p-values below 0.05 were defined as significantly differentially methylated between the different conditions. Integrative Genome Viewer (IGV) was used to visualise the DMS. Principal component analysis (PCA) and plot heatmaps were computed using the entire set of significantly differentially methylated CpGs between different conditions.

### Pathway analysis

Pathway analysis was performed using MetaCore software (Thomson Reuters) version 6.31 using the process networks gene ontologies default analysis in differential methylated CpGs of each comparison.

### Bisulfite Pyrosequencing

Genomic DNA was prepared from fibroblasts using the QIAamp DNA Blood Mini kit (Qiagen). The DNA (1 µg) was bisulfite modified using the EpiTect bisulfite kit (Qiagen). A PCR amplification of bisulfite modified DNA (2 µl) were performed using the Pyromark PCR kit (Qiagen). The PCR program was 95 °C 4 min; 95 °C 30 s, 56 °C 30 s, 72 °C 2 min x 35 cycles; 72 °C 4 min. We use the HS-SHROOM1_06_PM pyromark primers (Qiagen).The PCR products were visualised with agarose gel electophoresis. Then, they were directly sequenced using the PyroMark Q48 Autoprep (Qiagen) according to the manufacture instructions.

### 5-Azacytidine demethylation

EstRASF were cultured in the presence of 0.5, 1 and 2 μM 5-Azacytidine (5-AzaC) for 6 days. Then, total RNA was extracted with Miniprep kit (Qiagen) and reverse transcribed to cDNA with reverse transcriptase (Applied Biosystems). Quantitative PCR reaction was performed with SYBR green (Roche) on Taqman 7500 Real-time PCR system (Applied Biosystems). Exon spanning specific primers for DNMT1 FW 5′TAACAGAAAAGGAATGTGTGAAGG3′ REV 5′TATTTCTGTTTGCAGAAATTCGTGC3′ and TET1 FW 5′TGCACCCTCAATGAAAATCGT3′- REV 5′GGGCTTGGGCTTCTACCAAA3′ were used. Cycle thresholds (Ct) were normalized to the housekeeping gene RPLO.

### Data Availability

The datasets generated during and/or analysed during the current study are available with the ArrayExpress accession code: E-MTAB-6581 and E-MTAB-6582.

## Electronic supplementary material

Supplementary information

Dataset 1

Dataset 2

Dataset 3
