# Supplementary material for: Analysis of early changes in DNA methylation in synovial fibroblasts of RA patients before diagnosis
Source: Sci Rep. 2018 May 9;8:7370. doi: 10.1038/s41598-018-24240-2 (PMC5943364; doi:10.1038/s41598-018-24240-2)
Supplement: Supplementary file 1 — Supplementary information [file 41598_2018_24240_MOESM1_ESM.pdf]

Analysis of early changes in DNA methylation in synovial fibroblasts of RA patients before diagnosis

Emmanuel Karouzakis[1*], **Karim Raza**[2,4], **Christoph Kolling**[3], **Christopher D. Buckley**[2], **Steffen Gay**[1], **Andrew Filer**[2,5+], **Caroline Ospelt**[1+]

[1]Center of Experimental Rheumatology, Department of Rheumatology, University of Zurich, Zurich, CH-8952, Switzerland

[2]Institute of Inflammation and Ageing, University of Birmingham, Edgbaston, B15 2TT, UK

[3]Schulthess Clinic, Zurich, CH-8008, Switzerland

[4]Sandwell and West Birmingham Hospitals NHS Trust, West Bromwich, B71 4HJ, UK

[5]University Hospitals Birmingham NHS Foundation Trust, Birmingham, B15 2GW

*emmanuel.karouzakis@usz.ch

[+]These authors contributed equally

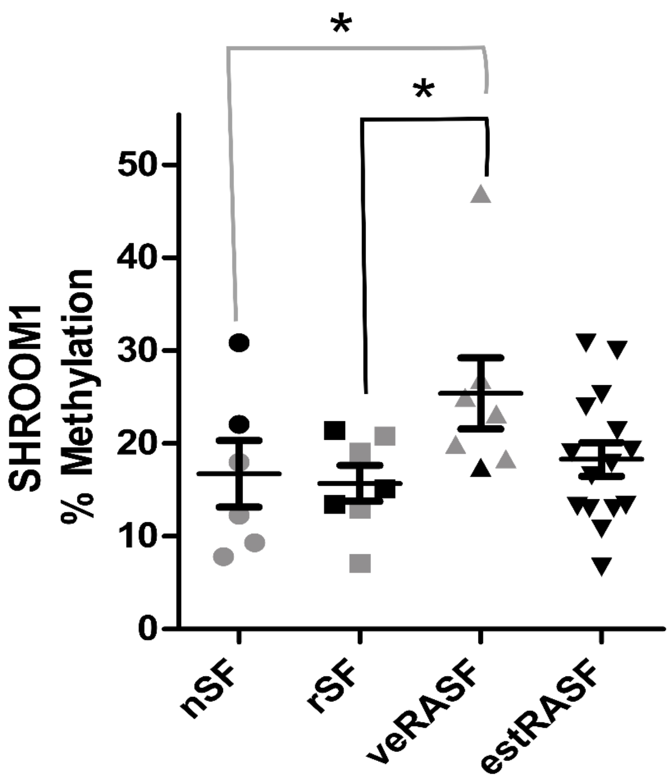

Supplementary Figure 1: Array validation of SHROOM1 methylation by bisulfite pyrosequencing. SHROOM1 promoter methylation is hypermethylated in the veRASF of the array patients (gray marks). Validation of DNA methylation of SHROOM1 in new samples (black marks) from normal (,n=6), rSF (n=7), veRASF (n=7) and estRASF(n=15) patients. Nonparametric Mann-Whitney test, *p<0.05, in gray statistical analysis only with array patients and black includes the addition of the new samples.

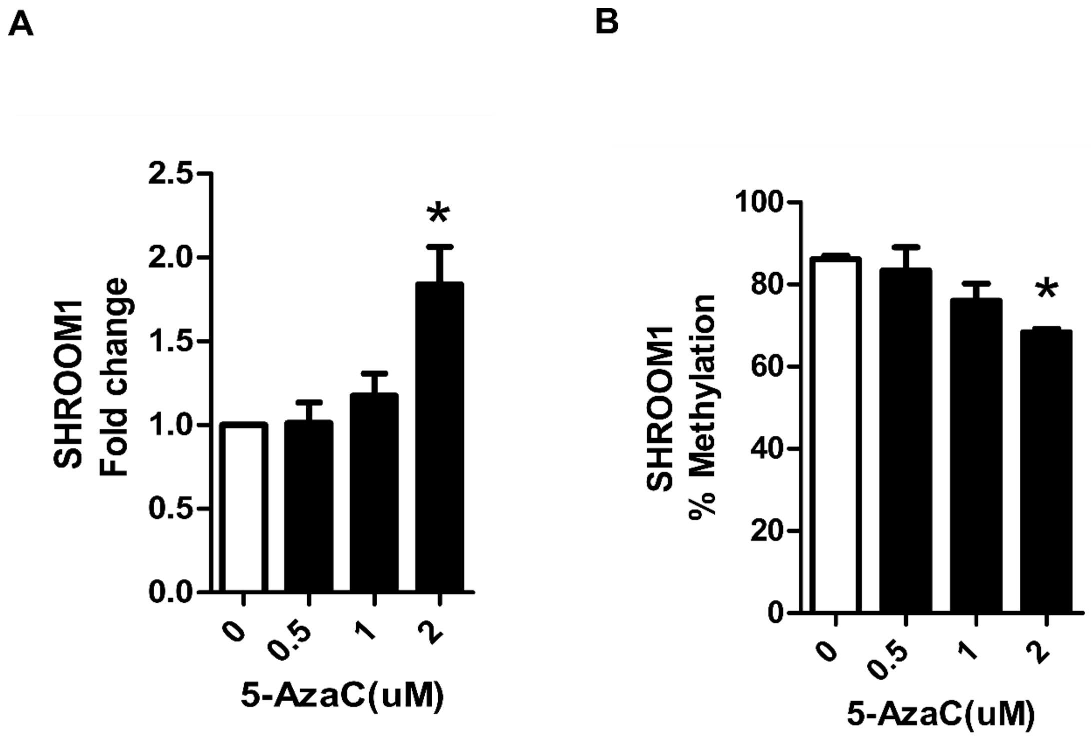

Supplementary Figure 2: Transcript expression and DNA methylation analysis of SHROOM1 in estRASF cultures treated with different concentrations of 5-azacytidine (5-AzaC). A) SHROOM1 mRNA expression is upregulated during 5-AzaC treatment in estRASF (n=4) B) Bisulfite pyrosequencing of SHROOM1 promoter showed a decrease in the methylation levels during 5-AzaC treatment (n=2). Student paired T-test,*p<0.05.

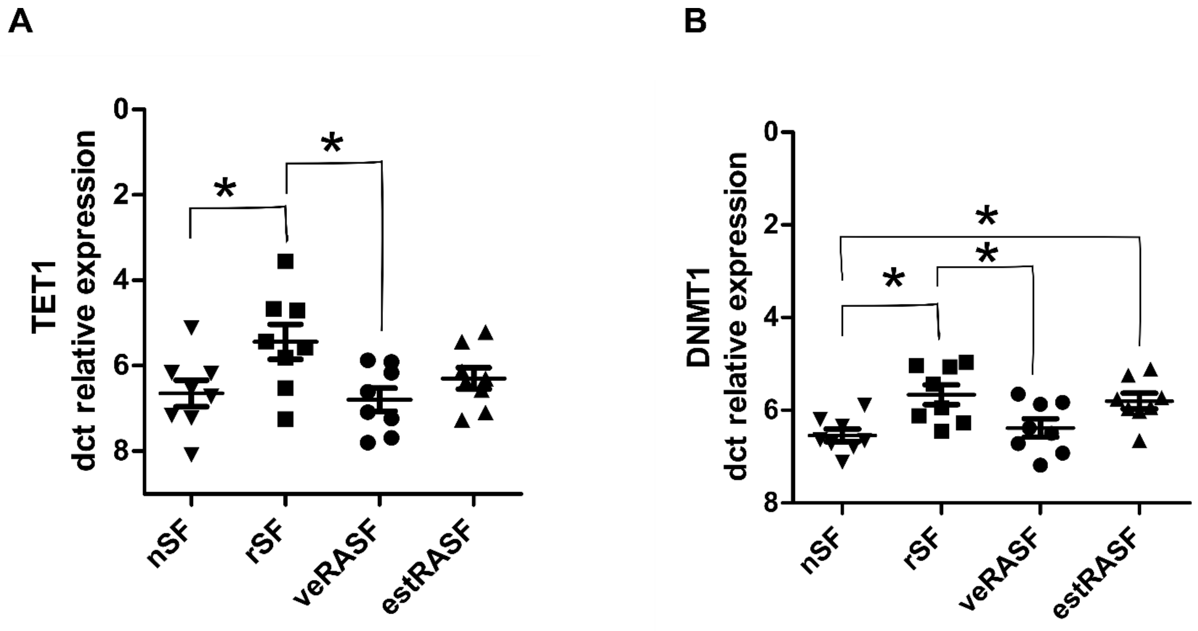

Supplementary Figure 3: Transcript expression of TET1 and DNMT1 between the different disease groups. TET1 (A) and DNMT1 (B) mRNA is significantly increased in rSF in comparison to the normal group and to veRASF. Nonparametric Mann- Whitney test, *p<0.05.

sample	diagnosis	joint	ccp pos/neg	rhf pos/neg	age	sex	disease duration	das28 baseline	CRP (mg/l)	DMARD
1	arthralgia	knee	n	n	44	M	NA	NA	NA	none
2	arthralgia	knee	n	n	38	M	NA	NA	NA	none
3	arthralgia	knee	n	n	34	M	NA	NA	NA	none
4	arthralgia	knee	n	n	38	F	NA	NA	NA	none
5	Parvovirus	knee	n	n	40	F	4 wks	3.9	0	none
6	Parvovirus	knee	n	n	45	F	1 wk	4.0	0	none
7	reA	knee	n	n	32	M	7 wks	2.9	10	none
8	unclassified	knee	n	n	64	M	6 wks	4.5	15	none
9	unclassified	knee	n	n	33	M	4 wks	6.7	14	none
10	unclassified	knee	n	n	72	M	8 wks	3.6	0	none
11	unclassified	knee	n	n	32	M	10 wks	3.7	0	none
12	VeRA	knee	n	n	70	F	5 wks	6.0	26	none
13	VeRA	knee	n	n	45	F	10 wks	3.8	12	none
14	VeRA	knee	n	n	63	F	4 wks	5.1	9	none
15	VeRA	knee	n	n	48	F	2 wks	3.5	102	none
16	VeRA	ankle	p	p	42	F	2 wks	8.3	40	none
17	estRA	knee	p	p	54	F	16 years	NA	47	leflunomide, adalimumab, corticosteroide
18	estRA	knee	p	p	64	F	7 years	1.9	2	adalimumab, methotrexate
19	estRA	knee	p	p	70	F	50 years	NA	NA	leflunomide, sulfasalazine, corticosteroide

ccp = anti-citrullinated peptide antibodies (<20 U/ml = negative); rhf = rheumatoid factor (<14 U/ml = negative); CRP = c-reactive protein; DMARD = disease modifying antirheumatic drugs; NA = not assessed; wks = weeks; reA = reactive arthritis

Supplementary Table 1: Patient`s characteristics.
